# Modeling Interval Timing by Recurrent Neural Nets

**DOI:** 10.3389/fnint.2019.00046

**Published:** 2019-08-28

**Authors:** Theodore Raphan, Eugene Dorokhin, Andrew R. Delamater

**Affiliations:** ^1^Institute for Neural and Intelligent Systems, Department of Computer and Information Science, Brooklyn College of City University of New York, Brooklyn, NY, United States; ^2^Ph.D. Program in Computer Science, Graduate Center of City University of New York, New York, NY, United States; ^3^Ph.D. Program in Psychology and Neuroscience, Graduate Center of City University of New York, New York, NY, United States; ^4^Department of Psychology, Brooklyn College of City University of New York, Brooklyn, NY, United States

**Keywords:** temporal coding, perception of time, interval timing, temporal averaging, peak procedure

## Abstract

The purpose of this study was to take a new approach in showing how the central nervous system might encode time at the supra-second level using recurrent neural nets (RNNs). This approach utilizes units with a delayed feedback, whose feedback weight determines the temporal properties of specific neurons in the network architecture. When these feedback neurons are coupled, they form a multilayered dynamical system that can be used to model temporal responses to steps of input in multidimensional systems. The timing network was implemented using separate recurrent “Go” and “No-Go” neural processing units to process an individual stimulus indicating the time of reward availability. Outputs from these distinct units on each time step are converted to a pulse reflecting a weighted sum of the separate Go and No-Go signals. This output pulse then drives an integrator unit, whose feedback weight and input weights shape the pulse distribution. This system was used to model empirical data from rodents performing in an instrumental “peak interval timing” task for two stimuli, Tone and Flash. For each of these stimuli, reward availability was signaled after different times from stimulus onset during training. Rodent performance was assessed on non-rewarded trials, following training, with each stimulus tested individually and simultaneously in a stimulus compound. The associated weights in the Go/No-Go network were trained using experimental data showing the mean distribution of bar press rates across an 80 s period in which a tone stimulus signaled reward after 5 s and a flash stimulus after 30 s from stimulus onset. Different Go/No-Go systems were used for each stimulus, but the weighted output of each fed into a final recurrent integrator unit, whose weights were unmodifiable. The recurrent neural net (RNN) model was implemented using Matlab and Matlab’s machine learning tools were utilized to train the network using the data from non-rewarded trials. The neural net output accurately fit the temporal distribution of tone and flash-initiated bar press data. Furthermore, a “Temporal Averaging” effect was also obtained when the flash and tone stimuli were combined. These results indicated that the system combining tone and flash responses were not superposed as in a linear system, but that there was a non-linearity, which interacted between tone and flash. In order to achieve an accurate fit to the empirical averaging data it was necessary to implement non-linear “saliency functions” that limited the output signal of each stimulus to the final integrator when the other was co-present. The model suggests that the central nervous system encodes timing generation as a dynamical system whose timing properties are embedded in the connection weights of the system. In this way, event timing is coded similar to the way other sensory-motor systems, such as the vestibulo-ocular and optokinetic systems, which combine sensory inputs from the vestibular and visual systems to generate the temporal aspects of compensatory eye movements.

## Introduction

The temporal encoding of significant events in neural structures has become an important consideration in a wide range of adaptive behaviors. The importance of time estimation in humans was first described by François (1927), but publicized by Hoagland (1933, 1935), when he noticed that his wife counted from 1 to 60 at an estimated 1 count/sec differently when her temperature changed as a result of influenza (Hoagland, 1935). Other studies on interval timing showed that a visual stimulus associated with a standard duration could be identified amongst other intervals that were given to subjects (Wearden et al., 1997). This work showed that this estimation of time intervals could be scaled for standard deviation as the interval was increased according to Weber’s law (Wearden et al., 1997).

Following Pavlov’s work on conditioning (Pavlov, 1927) and subsequent work on operant conditioning (Skinner, 1938, 1951), there has been growing interest in studying how animals learn to time the arrival of key events, such as reward (Staddon and Higa, 1999; Staddon, 2005). In a typical experiment, for instance, a stimulus might be presented and in its presence food reward is made available for the first response that occurs after *t* seconds have elapsed. At issue is how the animal encodes the time at which food becomes available and then distributes its behavior accordingly.

Research on interval timing has generated a variety of different theoretical models, the most popular of which is based on an internal “pacemaker-accumulator theory” (Treisman, 1963; Church, 1978) also known as scalar expectancy theory (SET) (Gibbon, 1977). According to this “clock model” (Gibbon, 1977; Gibbon et al., 1984), the onset of a signal closes a switch that gates pulses to an accumulator until a reinforcement signal ends the accumulation of pulses that are stored in a reference memory. This accumulation of the number of stored pulses establishes a distribution of values related to the reinforced duration. On subsequent trials the signal causes retrieval of a value from reference memory, and responding is based on a discrepancy rule and a decision threshold. The difference between the current accumulated time (working memory) and the reference memory value is constantly updated, and responding is predicted to occur when the ratio of that difference to the reference memory value falls below some decision threshold. As trial time elapses, the relative difference decreases and the probability of responding increases. However, if reinforcement is omitted and the trial signal remains beyond the expected time of reinforcement, the relative discrepancy grows and responding decreases again. This pattern of responding is typically observed in empirical studies when averaging responses across many individual trials in a task known as the “peak procedure” (Catania, 1970; Roberts, 1981).

Several other authors have objected to the pacemaker-clock approach to interval timing and have proposed alternative frameworks (Killeen and Fetterman, 1988; Grossberg and Schmajuk, 1989; Church and Broadbent, 1990; Machado, 1997; Staddon and Higa, 1999; Staddon, 2002, 2005; Matell and Meck, 2004; Oprisan and Buhusi, 2011; Buhusi and Oprisan, 2013). Several of these alternative approaches rest on the notion that as time passes from the onset of a stimulus, processing initiated by that stimulus undergoes a series of discriminable states and that the dominantly active state at the moment of reward becomes strengthened. In this way, learned behavior can be said to be “timed.” This notion was, perhaps, first noted by Pavlov (1927, p. 104) in his attempt to explain the phenomenon of “inhibition of delay”:

“*nerve cells which are being excited pass through a series of successive physiological changes. In accordance with this it is obvious that if a definite unconditioned reflex is repeatedly evoked coincidently with any one particular physiological state of the cerebral cells, it is this definite state and no other that acquires a definite conditioned significance.”*

The idea expressed by Pavlov has been formalized in a variety of ways. For example, Staddon and Higa (1999) introduced a “multiple time scale model” of habituation as the basis of interval timing, Grossberg and Schmajuk (1989) introduced a “spectral timing” approach, and Killeen and Fetterman (1988) and Machado (1997) assumed a series of stimulus-initiated behavioral states as the basis of temporal control. In any given conditioning trial, once that particular state associated with reward is re-entered then responding will arise.

A third class of theories has also been developed to explain interval timing. Church and Broadbent (1990) introduced a connectionist model and Matell and Meck (2004) and Oprisan and Buhusi (2011); also (Buhusi and Oprisan, 2013) extended this to a neural network equivalent – the “striatal beat theory.” The basic notion is that the brain contains multiple oscillators, i.e., neurons that fire with different periodicities, and time can be encoded as the unique oscillator firing pattern present at the moment of reward. Subsequently, when that firing pattern is approximated on a given conditioning trial, responding becomes more likely through a pattern matching decision process.

Another approach, using large interconnected neurons have also been applied to modeling certain fundamental aspects of interval timing behavior. One key feature of the timing system is that as the interval to be estimated increases, the variance of responding around that estimate also increases in accordance with Weber’s law. This is known as the “scalar timing” principle (Gibbon, 1977, 1992; Gibbon et al., 1984). The approach of using “large population clock neurons” that give rise to a diffusion equation whose drift rate is learned within these interconnected neurons was utilized to model scalar expectancy in peak timing experiments (Simen et al., 2011, 2013; Luzardo et al., 2017; Hardy and Buonomano, 2018). Different intervals are timed, in this model, by different proportions of neural populations that generate timing pulses engaged by the stimulus, with higher proportions effectively increasing the diffusion drift rate such that a response is triggered sooner.

One empirical phenomenon that has been especially challenging for all approaches is how multiple stimuli are combined to generate timing behavior. Matell and his colleagues have noted that when rodents are trained in a peak procedure with two separate stimuli indicating different intervals to time, responding to the stimulus compound reflects an averaging of the two intervals rather than memories for each trained interval (Swanton et al., 2009; Swanton and Matell, 2011; Matell and Henning, 2013; Matell and Kurti, 2014; Delamater and Nicolas, 2015; De Corte and Matell, 2016a, b). This result is problematic because most theories anticipate behavior to be controlled by each of the two intervals trained separately. For instance, first consider SET’s assumptions. When trained with different stimuli, reference memory should include one distribution of trained intervals appropriate for stimulus A and a second distribution for stimulus B. When a stimulus compound is presented, stimulus AB, one interval from each of the two reference memory distributions should be retrieved and responding should emerge whenever a working memory representation of elapsed time is close to each of those two intervals. In other words, there is no mechanism for responses to compound stimuli to reflect the average of the two intervals built into SET. Similarly, if timing were related to a series of discriminable states initiated by presentation of the stimulus, then, once again, whenever the system approached those two dominant states trained individually, responses should be maximal at each of those two times rather than at some intermediate time. The multiple oscillator and striatal beat theories would have similar difficulty because each reinforced activation pattern should govern responding, in a manner analogous to SET.

In this paper, we explore the use of a simple RNN model to predict interval timing. Our approach differs from others in several ways. First, we do not assume that a clock system is engaged to generate a steady stream of pulses. Rather, our RNN has a dynamic response to an input stimulus, which is modeled by a step function in Tone and Flash. The response is determined by input weights and recurrent feedback weights that are learned by a reinforcement signal at a specific time from stimulus onset. The weights of the RNN are stored in memory (i.e., in the network itself) but can be updated (learned) with repeated exposure to signal and reinforcement. Second, as developed in greater detail below, we assume that different recurrent processes adopt distinct “Go” and “No-Go” behavioral functions that summate within a “timing circuit” and this, ultimately, feeds into a final recurrent integrator output stage that governs the system’s response. Finally, we make the important additional assumption that when multiple stimuli are presented together (as in a temporal averaging study), interactions among the stimuli take place such that the effective “salience” of each stimulus is impacted by the other. On the basis of the temporal dynamics of the recurrent processes that make up the network, we show that temporally organized behaviors can be trained with empirical data obtained from rodents performing in a peak procedure. Thus, the final recurrent process with its learned weights generates a distribution of output that matches the response patterns of the animal. Furthermore, after training different recurrent systems with different intervals of reinforcement and different stimuli (such as a tone and flash), we show that dynamic interactions between these two systems can predict temporal averaging. This idea suggests that the encoding of interval timing is embedded in the connection weights of a relatively small RNN, without the need for a fixed internal clock (Staddon, 2005). In addition, by considering the recurrent interactions within and between neural processing units, other aspects of dynamic temporal control may be shown to emerge in empirically meaningful ways.

## Materials and Methods

### Experimental Data

#### Procedures

##### Subjects

Male and female Long–Evans rats (*n* = 8 of each) bred at Brooklyn College (from Charles River Labs descent) were housed in a colony room on a 14:10 light:dark schedule, and throughout the experiment were maintained at 85% of their free feeding weights (ranging from 223 to 263 g for females, and from 348 to 377 g for males). All procedures on these animals were approved by the IACUC of Brooklyn College, and were in compliance with NIH guidelines as identified in the *Guide for the Care and Use of Laboratory Animals, 8^th^ Ed*.

##### Preliminary training

The rats first learned to retrieve food pellets (45 mg TestDiet 5TUM, 45 mg Bio-Serv #50021) from a recessed “food magazine” (3.0 cm × 3.6 cm × 2.0 cm, length × width × depth) located on the front wall of a rectangular shaped conditioning chamber (BRS Foringer RC series, measuring 30.5 cm × 24.0 cm × 25.0 cm, length × width × height). These chambers were housed inside separate sound- and light-resistant shells (Med Associates, ENV-022V). During each of 2 days, the rats were placed in the conditioning chambers for 2, 20-min sessions. In each session, one of the two pellet types was delivered to the food magazine 20 times at random, with the order counterbalanced. A response lever (4.0 cm in width) was located 3.0 cm to the left of the magazine and 8.0 cm above the chamber floor (that consisted of steel rods spaced 2.0 cm apart). On the next day the rats learned to press this response lever to obtain food reward, until 30 rewards of each type were earned.

##### Peak procedure

Over the next 40 days the rats were trained on the “peak procedure.” In each training session, the rats learned to obtain reward for the first lever press response occurring after 5 s from the onset of an 80-s tone stimulus (Med Associate sonalert, 2900 Hz and 6 dB above background level of 74 dB provided by a ventilation fan), and 30 s from the onset of an 80-s flashing light stimulus. These stimulus-interval assignments were not counterbalanced because prior research has shown that temporal averaging effects are more likely to occur with the present assignments (Swanton and Matell, 2011; Matell and Henning, 2013; Matell and Kurti, 2014). Two 28 volt light bulbs were used for this purpose and these were located in the top of the rear wall of the chamber opposite the food magazine, behind a translucent plastic sheet used to protect the bulbs and diffuse the light. The lights flashed at a frequency of 2/s with equal on-off pulses. The chamber was dark otherwise. In each training session, there were 16 conditioning trials with the tone stimulus and 16 with the flash stimulus. The inter-trial interval (i.e., time from offset of one stimulus to onset of the next) was 40 s in each of the first two sessions, 80 s in the next two sessions, and 120 s in each session thereafter. The 40 training sessions were arranged in “blocks” of four training sessions. In each of the first two blocks of training, reinforcement was made available for the first lever press response occurring after the critical time interval on a predetermined 75% of the tone and flash trials. In the next three training blocks, reinforcement was made available on 25% of the tone and flash trials. Thereafter, reinforcement was made available on 25% of the tone trials and 75% of the flash trials (in order to maintain comparable peak levels of responding on each trial type). Importantly, the non-reinforced tone and flash trials were regarded as “probe” trials in which lever press responding was assessed in 1-s intervals, starting 20 s prior to stimulus onset and extending for the entire 80 s of stimulus presentation. In each conditioning session the order of these reinforced and non-reinforced probe trials was randomly determined.

##### Temporal averaging assessment

The same procedures continued in the 11^th^ block of training as in the 10^th^ block. However, four additional non-reinforced “probe” trials occurred in which the tone and flash stimuli were presented as a simultaneous stimulus compound. Responding on each of these non-reinforced probe trials with tone, flash, and the tone + flash compound constituted the main data of interest.

## Results

### Experimental Results

**FIGURE 1:**
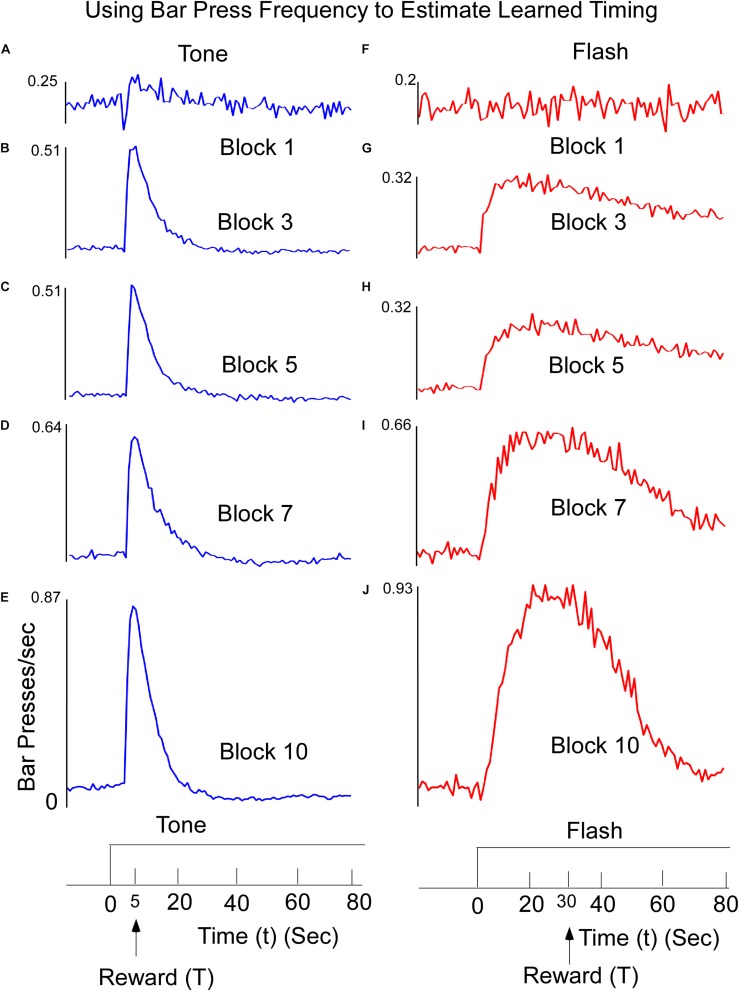
Learned reward timing on tone **(A–E)** trials. Animals were trained to press a Bar to receive a food reward at T = 5 s following onset of a 60-s Tone stimulus. Animals received the reward for the first lever press after this time. Interspersed throughout training, rats were also tested on non-reinforced “probe” trials to determine how responding was temporally distributed within the trial. Bar Press frequency was computed by counting the number of Bar Presses within a 1 s window. The peak Bar Press/sec was computed and increased rapidly and then declined. The width of the function describing the Bar press frequency decreased as a function of training, but the time to reach the peak stayed relatively constant. The data are presented across representative 4-session Blocks of training. **(F–J)** Learned timing for Flash input. Animals received the food reward for the first lever press occurring after T = 30 s. The time to reach peak Bar Press frequency was longer, but as the animals learned, the width of the function decreased.

Mean lever-presses per second on non-reinforced tone and flash probe trials were recorded in 1-s time bins for the 20-s period preceding stimulus onset and for the entire 80-s stimulus period. The data were averaged across trials and days for an individual subject and across rats for each 4-session block of training. The response functions on both Tone (Figures 1A–E) and Flash (Figures 1F–J) trials were progressively shaped over the course of training, with responding peaking increasingly closer to the anticipated reward times as training proceeded. There were three noteworthy differences between tone and flash responses by the end of training. First, in the presence of the Tone, the response rapidly rose and peaked close to the anticipated time of food availability (5 s) and declined rapidly thereafter (Figure 1E). In the presence of the Flash stimulus, the peak response occurred close to the anticipated time of food availability (30 s) but it rose to that peak value more slowly than for Tone, before declining gradually thereafter and more slowly than for Tone. Second, these differential patterns of responding emerged more quickly over training to the Tone stimulus than the Flash (Compare Figures 1A–E with Figures 1F–J). Third, while the responses in both stimuli rose more rapidly than they declined, the overall variability in responding (e.g., the width of the response distributions) was larger for the flash than for the tone stimulus, a fact that is likely due to the length of the time interval to be estimated. It is also noteworthy that by the end of training, responding to the Tone stimulus decreased to below-baseline (i.e., pre stimulus) levels. This suggests that the stimulus actively inhibited lever press responding late in the Tone stimulus, periods in which food was never presented but were clearly differentiated from the early periods in which food was frequently available. Such behavior was not apparent to the Flash stimulus.

**FIGURE 2:**
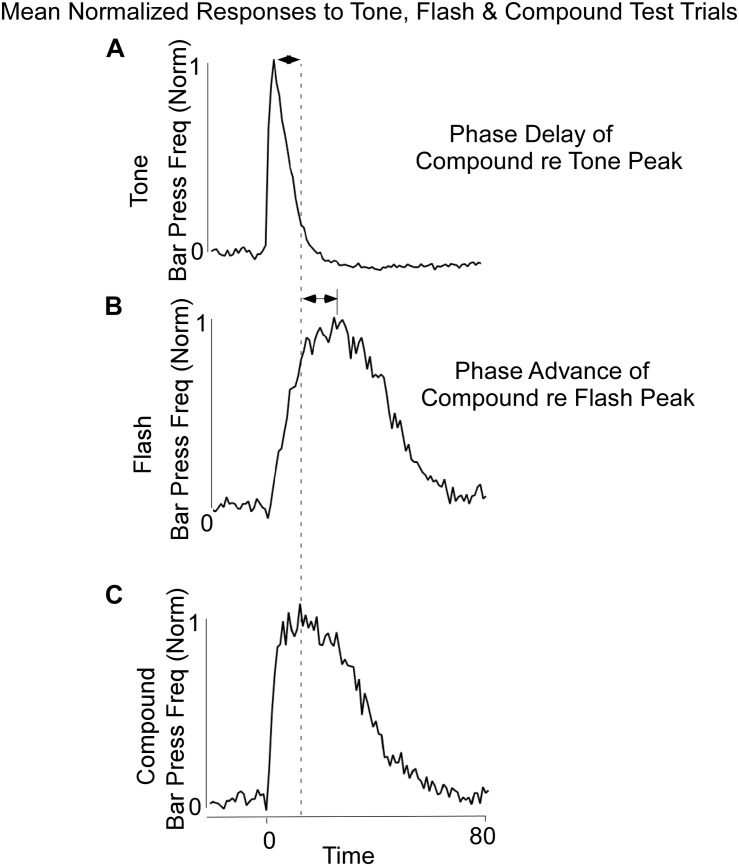
Average Bar Press frequency on non-reinforced probe trials for Tone **(A)**, Flash **(B)**, and Combined Tone + Flash **(C)** trials in Block 11 of training. There was a phase delay of the Combined Tone + Flash relative to Tone alone trials and a Phase advance of the Combined Tone + Flash relative to Flash alone trials. Thus, the Combined Tone + Flash stimulus generated a response function whose peak and variance were between those observed on Tone and Flash trials. Whereas the peak of the Combined Tone + Flash function was somewhat closer to Tone alone, its width was closer to that seen to Flash alone.

Lever press responses during Block 11 included non-reinforced probe trials with the Tone, Flash, and Tone + Flash stimuli. For the purposes of analysis, the data were normalized in each 1-s time bin by expressing response rate as a proportion of maximal response rate. The maximal response rates on each trial type averaged across animals did not appreciably differ (mean maximal response rates on Tone, Flash, and Tone + Flash trials, respectively, were 1.00, 0.96, and 0.98 responses/s). The normalization was done for each of the three (3) cues separately and the slightly different rates reflect the differing peak times across animals. However, when peak rates were computed for each individual animal, the maximal response rate on tone trials (mean = 1.10 responses/sec) was significantly less than on flash trials (mean = 1.28 responses/sec) and on compound trials (mean = 1.23) trials [*F*(2,28) = 4.84, *p* < 0.05]. The model simulations were compared to the response rates averaged across animals. Once again, differential responses were observed to the Tone and Flash stimuli, when tested individually, with earlier and less variable responses to Tone (Figure 2A) than to Flash (Figure 2B). Importantly, responses to the Tone + Flash compound stimulus reflected an “averaging” of the two individual functions with a phase delay with reference to Tone alone and phase advance with reference to Flash alone. Notably, it did not result in peaks occurring at 5 and 30 s. This indicates that there is an integrative dynamic process that combines the responses to the stimuli rather than a simple summation of the two individual response functions.

To explore these data quantitatively we first evaluated the response functions on these three trial types using the curve-fitting method of Matell and Kurti (2014). The normalized lever press data on Tone, Flash and Compound trials for each animal were fit with a dual asymmetric sigmoid function, using the curve fitting package in Matlab, Cambridge, MA, United States. The time at which the function reached a peak (Peak Time) was then determined for the 16 animals for Tone, Flash and Compound stimuli. The width of the function was determined as the time between values that were reduced by 50% from the peak value of the function (Width) (Matell and Kurti, 2014). These values as well as their averages over all animals are shown in Table 1. The data from one animal was not included in this analysis because response rates on Flash and Compound trials did not display a clear peak function.

**TABLE 1:** Each rat had 10 blocks of training.

**Rat #**	**Peak time (PT)**	**Width 50% (W-50)**	**Ratio PT/W-50**
**Flash**			
1	21.75	50.19	0.4332
2	25.14	38.73	0.6489
3	22.21	37.78	0.5877
4	27.98	35.94	0.7784
5	N/A	N/A	N/A
6	26.34	50.34	0.5231
7	30.68	37.70	0.8137
8	25.69	43.35	0.5925
9	26.26	34.27	0.7660
10	28.86	44.08	0.6545
11	30.99	43.33	0.7152
12	25.00	43.56	0.5738
13	24.06	36.58	0.6575
14	27.21	32.14	0.8466
15	25.78	45.97	0.5607
16	24.22	34.99	0.6920
Avg.	26.14	40.60	0.6563

**Tone**			
1	5.93	10.38	0.5711
2	6.22	12.70	0.4893
3	4.17	6.87	0.6066
4	4.02	6.56	0.6126
5	N/A	N/A	N/A
6	5.04	8.33	0.6046
7	5.03	7.90	0.6363
8	4.67	9.83	0.4746
9	4.60	7.37	0.6240
10	4.44	14.85	0.2988
11	3.68	10.80	0.3406
12	4.06	7.53	0.5384
13	4.30	7.80	0.5510
14	4.51	8.39	0.5370
15	4.99	7.22	0.6905
16	5.24	10.40	0.5036
Avg.	4.72	9.13	0.5225

**Compound**			
1	12.76	45.64	0.2795
2	19.06	34.81	0.5474
3	14.8	35.54	0.4164
4	14.41	34.57	0.4168
5	N/A	N/A	N/A
6	13.99	33.94	0.4121
7	12.49	28.17	0.4432
8	19.12	39.51	0.4839
9	17.56	36.42	0.4821
10	17.49	37.93	0.4610
11	16.14	36.64	0.4404
12	15.09	39.16	0.3853
13	15.77	33.54	0.4700
14	19.39	27.90	0.6947
15	20.23	38.36	0.5272
16	14.91	36.32	0.4104
Avg.	16.21	35.90	0.4580

*The Compound Probe sessions were contained in Block 11 and comprised non-reinforced presentations of flash, tone, and compound trials. The peak Time (PT) and Width between 50% of peak times (W-50) was obtained using the method of curve fitting used by Matell and Kurti (2014). The ratio of the peak time to the Width (W-50) using the method of Matell and Kurti (2014) could then be used as a measure of scalar invariance.*

First, we observed significant differences in peak times on these three trial types [means (±SEM) = 4.7 (0.18), 26.1 (0.70), and 16.2 (0.64) for Tone, Flash, and Compound, respectively], *F*(2,28) = 366.45, *p* < 0.000001. *Post hoc* tests using the method of Rodger (1974) confirmed that peak times on each trial type differed from one another with the peak times for Flash > Compound > Tone, and with the peak time for Compound approximating the arithmetic average of the peak times for Tone and Flash.

Second, we computed the coefficients of variation (CV, width/peak time) for each animal for Tone, Flash and Compound trials in order to determine if responding was scalar invariant (Gibbon, 1977; Church et al., 1994). The mean CVs significantly differed for Tone, Flash, and Compound trials [means, respectively, = 1.95 (± 0.13), 1.57 (± 0.70), 2.26 (± 0.12), *F*(2,28) = 10.38, *p* < 0.0005], and *post hoc* tests confirmed that the three trial types differed from one another and were ordered as follows: Compound > Tone > Flash. This indicates that although the widths of the response functions increased with the interval to be timed, they did not do so proportionally with peak time (Table 1).

### Neural Net Modeling

**FIGURE 3:**
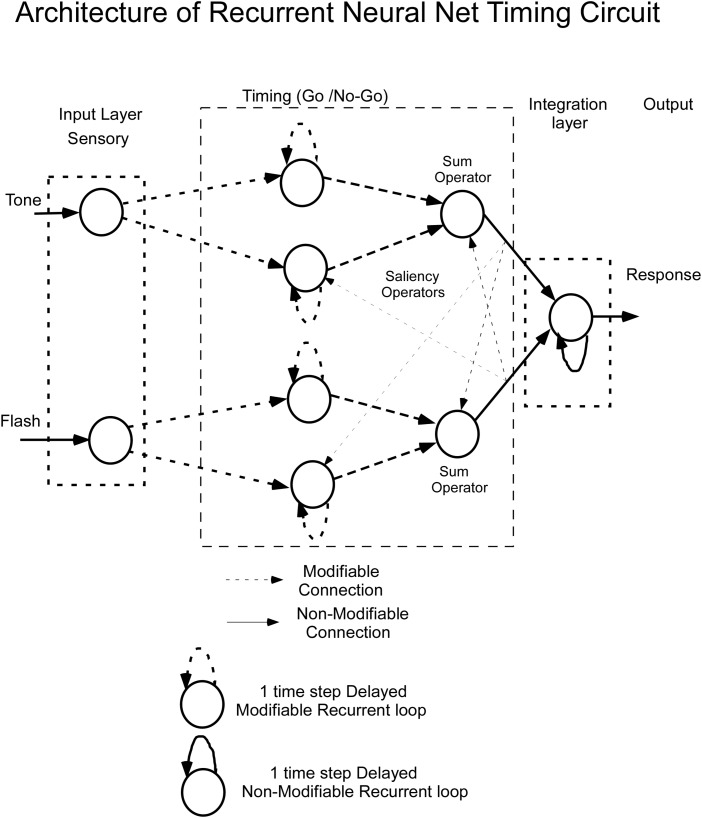
Architecture of the recurrent neural net (RNN) for timing learning. See text for details.

Bar press distributions for flash and tone inputs were conceptually modeled by using a neural net that has dynamic properties, whose dynamics, i.e., speed of response, are associated with specific nodes that have recurrent feedback loops (Figure 3). The input layer comprises separate sensory units, which code auditory and visual stimuli, denoted by Tone and Flash inputs. Output from these units enter the “Timing” component of the network, which consists of two recurrent units for each sensory input. These recurrent units are conceptualized as executing “Go” and “No-Go” behavioral functions. Outputs from these Go/No-Go units feed into a “Sum Operator” unit for the Tone and Flash inputs when presented individually (Figure 3). The summation of the Go/No-Go units implement a second order dynamical system with short and long time constants whose outputs oppose each other. A second order system has been useful in modeling the semicircular canal dynamics of the vestibulo-ocular reflex, which then activates a central velocity storage integrator (Raphan et al., 1979). These early modeling approaches motivated the development of the RNN presented in this study. A parsimonious feature of this approach is that there is a minimal number of neural processing units, each behaving as an integrator with a different time constant, can be used to model the response to constant tone, flash and compound stimuli. Approaches using large populations of neurons have also been used to model interval timing (Hardy and Buonomano, 2018). An approach that uses a single integrator driven by a bistable input layer consisting of a population of units generating a stochastic ramp and trigger (SRT) has been shown to generate a drift-diffusion model that simulates scalar invariance in interval timing (Simen et al., 2011). We found that we could better fit the compound test data in this study by using a piecewise linear activation function in the Sum Operator unit that had a linear part that saturated with too much or too little input. It was also required to implement a saliency operator, which was implemented by cross coupling between the two stimuli such that their effective “saliency” is diminished when presented together (see Saliency Operator Description below). All of the weights connecting nodes up to this point in the network were assumed to be modifiable, so they could learn the timing characteristics associated with the data. Output from the Tone and Flash summation units then feed into a final recurrent integrator unit that uses a linear activation function [f(x) = x]. Output from this integrator unit serves as the basis of the model’s performance. We now consider the details of the RNN and how learning was implemented to simulate the data across these two modalities.

### Recurrent Neural Network Model for Generating Interval Timing

The model was implemented using Matlab’s Neural Net ToolBox, and learning of the weights was obtained using Matlab’s machine learning libraries. The simulated data were generated from the model as output activation vectors corresponding to the 20 pre-stimulus and 80 stimulus time steps for each stimulus (Tone, Flash), and the mean lever press response data (normalized to maximal response rates, see Figure 2) for a particular stimulus were used to train the weights to minimize the mean square error of the comparison.

**FIGURE 4:**
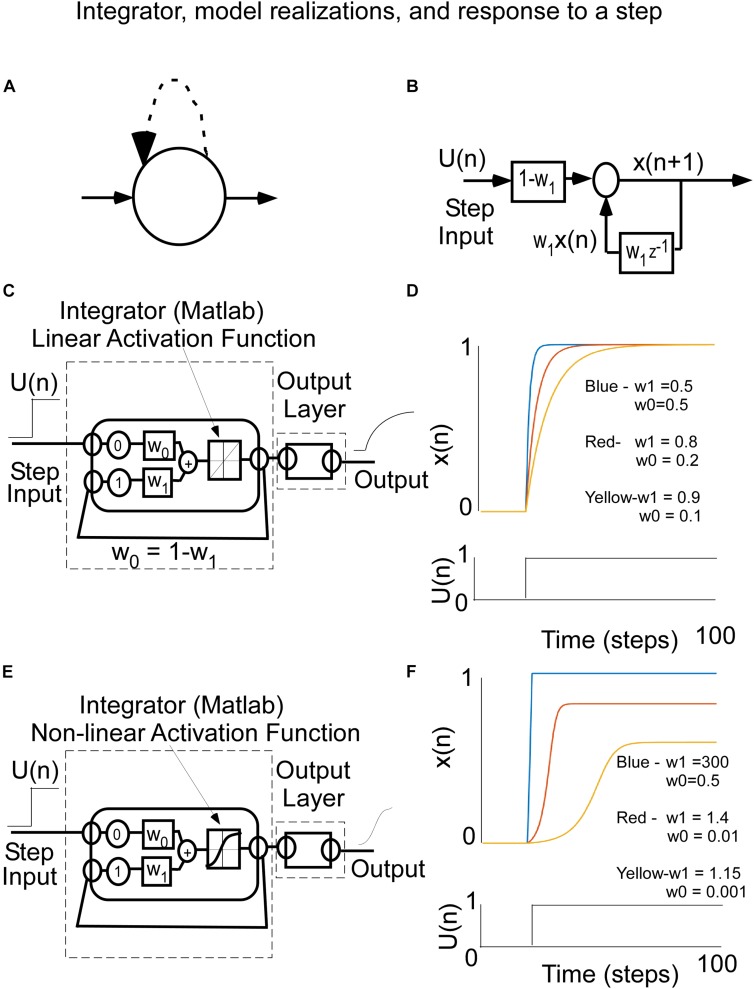
**(A)** Recurrent Node representation for a simple integrator. **(B)** Control theoretic input–output representation of an integrator The input in U(n) and the output is the state of the integrator. The w_1_ is the feedback (recurrent) weight and z^–1^ represents a one time step delay. The feedforward weight is W_0_. When W_0_ = 1-W_1_, the state x(n) rises to a value 1 with a rise time that is that is related to W_1__._ **(C)** Matlab Implementation of the integrator with a linear activation function. **(D)** Response of the integrator to a step of input for different weights, w_0_ and w_1_. Note that the closer the weight w_1_, which is the recurrent feedback weight, gets to 1, the longer the rise time to its steady state value. **(E)** Matlab implementation of the integrator with a squashing activation function [tanh(⋅)]. **(F)** This leads to a wider range of weights that produce different types of rise behavior. The larger the recurrent weight, the faster the response is to the steady state.

We first consider how a single recurrent loop implements timing (Figure 4A) and sheds light on the solution to the overall timing problem by combining multiples of these recurrent loops to implement a dynamical system. A single recurrent neural processing unit can be described analytically by a feedback loop, which is delayed by a single time step, represented by z^–1^, and a feedback weight, w_1_ (Figure 4B). This has been described in the system theory literature as an *integrator* (Zadeh and Desoer, 1963). The concept of an integrator has been utilized in modeling a wide range of phenomena related to the saccadic system (Raphan and Cohen, 1981; Robinson, 1981; Raphan, 1998), denoted by the velocity-position integrator, which has played an important role in transforming the velocity commands generated centrally to the position commands that the eye muscles receive to hold the eyes (Robinson, 1981; Raphan, 1998; Seung et al., 2000). Additional integrators have been identified in the vestibulo-ocular reflex (Raphan et al., 1979; Raphan and Cohen, 1996, 2002), vestibulo-sympathetic system (Raphan et al., 2016), and locomotion system (Cho et al., 2006, 2010; Osaki et al., 2008). In some instances, our definition of “integrator” has also been referred to as a “leaky” or “impure” integrator (see also Seung et al., 2000; Simen et al., 2011), whereas a feedback weight of 1, is referred to simply as an integrator (Zadeh and Desoer, 1963; Seung et al., 2000).

The integrator can be defined by a difference equation, which naturally lends itself to implementation as a RNN. The state, x(n), denotes the current state of the integrator and x(n + 1) is the next state after 1 time step, which is updated by a dynamic process.

The integrator can be represented analytically as

(1) x⁢(n+1)=W⁢x1⁢(n)+W⁢U0⁢(n)

where x(0) is the initial value of the state and there is a linear activation function (Figure 4C). The weight, w_1_, is the recurrent feedback weight, which determines the rate at which the output rises to a steady state level. The feed-forward gain (W_0_), i.e., the weight connecting an input unit to the integrator unit, determines the asymptotic level to which the output rises in response to a step input, U(n). When the feed-forward gain, W_0__,_ is set to W_0_ = 1- W_1_, the asymptotic output level is equal to the input unit’s activation level, which depends on an activation function that is linear (Figure 4D). The rate at which that asymptote is achieved depends upon w_1_. In response to a step input (Figure 4D), this unit’s behavior, for a Matlab simulation, changes over time differently for three different feedback weight values (where w_1_ = 0.9, 0.8, 0.5, Figure 4D), illustrating its timing capability from a long time constant to a short time constant. Notice (1) that in all three cases, the asymptotic output level matches the input level, and (2) that the closer the feedback weight, w_1_, is to 1.0, the longer is the rise time to reach the steady state value.

When implementing neural nets, non-linear activation functions, such as squashed S functions are generally used to increase the flexibility of the learning (Winston, 1993). In the Matlab toolbox, this squashed S activation function is the tanh(•). The integrator can now be represented mathematically by the following equation:

(2) x(n+1)=tanh[w(x(n))1+w0u(n)]

where the output of the integrator is squashed before it is fed back. It is implemented in Matlab as shown in Figure 4E. The response to steps of input follow the slow, medium, and fast rise although the weights are now different because of the squashed S function that generates the next state x(n) (Figure 4F). Because the input and recurrent weights are not aligned, the asymptotes that the responses rise to are different.

**FIGURE 5:**
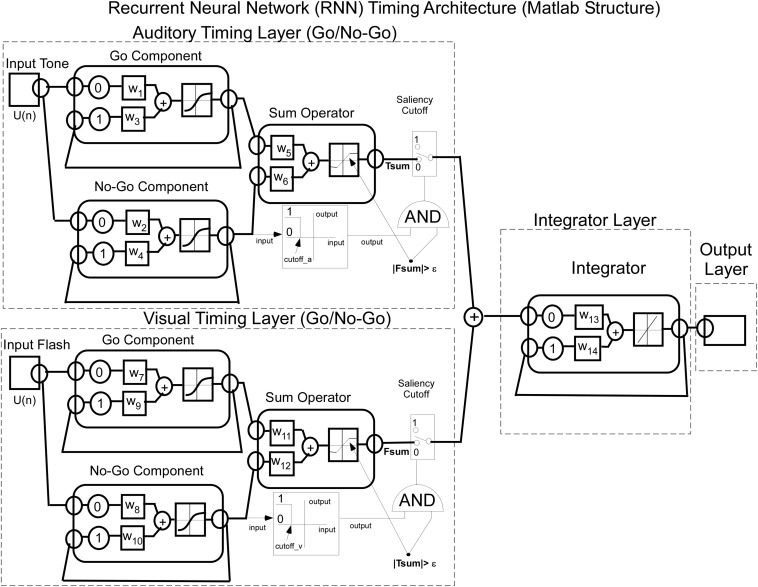
Matlab implementation of the RNN used to model interval timing. Note that each of the Tone and Flash stimulus inputs converge onto two distinct “timing” nodes (Go and No-Go) that adopt opposing fast and slow integrator behavioral functions with squashing activation function behavior. Output from these two timing nodes are then summed and are operated on by a saliency operator, which then adds their outputs and drives a fixed weight integrator.

The Matlab implementation of the RNN timing model for Tone and Flash stimulus inputs is a combination of integrators as shown in Figure 5. The delays in the feedback loops for recurrent units are denoted by having a 1 time step associated feedback path. This meant that each recurrent unit stimulated itself through weights, w_3_ – w_4_, w_9_ – w_10_, and w_14_.

It should be noted that the activation functions at each layer were different and suited for the intended purpose of the model. The first layer learned the weights from the data and a tanh function was used as the activation function for units at this layer. In the second layer (summator), the activation function was dependent upon whether one or two stimulus inputs were presented to the network. If a single stimulus was presented the activation function was linear, but if both stimuli were presented we used separate piecewise linear functions whose slopes, saturation and cutoffs differed for the two stimuli. As described in more detail below (Saliency Implementation), this was done in order to implement the idea that concurrently presented stimuli could interfere with one another’s processing, i.e., their “salience.” The final integrator was implemented with a linear activation function as its weight was fixed throughout the learning process. While the model explained the dominant features of the timing data, the saliency functions were necessary to more accurately fit the data for combined tone and flash compound tests (see Description and Equations implemented for Saliency, below).

### Model Equations Without Saliency

As explained above, the model is a dynamical system, which can be described as an interconnection of integrators.

For Tone input, the state update equations of the Go and No-Go units can be given as:

(3) T(n+1)Go=tanh[W3(T(n)Go)+W(U(n)Tone)1]

(4) T(n+1)NoGo=tanh[W(T(n)NoGo)4+W(U(n)Tone)2]

The initial state, T_Go_ (0) and T_NoGo_ (0) was assumed to be zero during the first 20 time steps, which was pre-stimulus state and the response was assumed to be due to the stimulus.

For Flash input, the state update equations of the Go and No-Go units can be given as:

(5) F(n+1)Go=tanh[W(F(n)Go)9+W(U(n)Flash)7]

(6) F(n+1)NoGo=tanh[W(F(n)NoGo)10+W(U(n)Flash)8]

For both Tone and Flash inputs, the Go and No-Go units feed into a Sum Operator (Figure 5).

When Tone and Flash responses are induced separately (no saliency), the Sum Operators have linear activation function and the update equations are given by:

(7) T(n)Sum=WT5(n)Go+WT6(n)NoGo

(8) F(n)Sum=WF11(n)Go+WF12(n)NoGo

Where n is the present time step and n + 1 is the next time step.

The summation is just used as input to the final Integrator whose equation is given by:

(9) x(n+1)=Wx14(n)+W[T(n)Sum+F(n)Sum]13

### Saliency Operator Equations

In this model, saliency is defined as the conditions that must be met when two (or more) stimuli are present for one stimulus to modify the transmission of the other stimulus. The saliency operation that we considered was based on the examination of the compound response as compared to the response predicted without saliency. One aspect of the compound response based on our data (Figure 2) was that it did not have an overshoot as was predicted by the model without saliency (see section Machine Learning for Combined Tone + Flash Trials below). This required the presence of a saturation effect to limit the overshoot.

Thus, the saliency operation in this model for Flash interfering with Tone processing is the saturating of the summed Tone Go/No-Go signal when the Flash signal is present. This is represented by a modification of the activation function in the Sum Operator for Tone by incorporating a saturation and a modification of the slope that produces T_Sum_ (Figure 5).

The implementation of saliency had bilateral effects, where not only does the Flash stimulus interfere with Tone’s processing, but the No-Go component of the Tone stimulus, can also affect Tones’s combined Go/No-Go processing.

With the saliency operator defined as above, the outputs of the Sum Operator for Tone when Flash is present are given by:

If

(10) WT5(n)Go+WT6(n)NoGo≥0

Then,

(11) T(n)Sum=Min[T(WT5(n)Go+WT6(n)NoGo)Sat,T]Sat

Similarly,

If

(12) WT5(n)Go+WT6(n)NoGo<0

Then,

(13) T(n)Sum=Max[T(WT5(n)Go+WT6(n)NoGo)Sat,-T]Sat

That is, if the weighted summation, T_Sum_(n), is less than zero, then the output of the summator is the maximum (Max) of the negative of Tone saturation (-T_Sat_) and the weighted summation scaled by T_Sat_. Thus, while negative signals are implemented, but in reality, both Tone and Flash are always positive.

Similar equations of saliency were implemented for Flash processing when being interfered with by Tone:

If

(14) WF11(n)Go+WF12(n)NoGo≥0

Then,

(15) F(n)Sum=Min[F(WF11(n)Go+WF12(n)NoGo)Sat,F]Sat

and,

If

(16) WF11(n)Go+WF12(n)NoGo<0

Then,

(17) F(n)Sum=Max[F(WF11(n)Go+WF12(n)NoGo)Sat,-F]Sat

Where T_Sat_ and F_Sat_ are the saturating values and slopes for the Sum Operator’s activation functions for Tone and Flash, respectively.

Because the decline in the compound response function was somewhat faster than that for Flash, it indicated that if there was a slight presence of the Tone Sum after a long time, i.e.,:

If

(18) |T|Sum>ε

Where ε is a value close to zero, then the Flash processing can still be modified by the activation of an AND gate together with the Flash No-Go component being less than **cutoff_v**, which outputs a 1 and the Flash is cutoff from activating the final integrator, i.e., the saliency cutoff switch is in the 1 position, and final integrator is just left to discharge without input from Flash processing.

Similarly, there is a modification of the process that produces T_Sum_ (Figure 5). If there is a presence of the Flash sum signal, i.e.,:

If

(19) |F|Sum>ε

and the Tone No-Go component is less than **cutoff_a**, then the output of its AND gate is 1 and the Tone processing is cutoff, i.e., the saliency cutoff switch is in the 1 position. The final integrator is similarly just left to discharge without input from Tone processing.

These components of saliency insure that there can be no activation of the final integrator with low level interference activations. These saliency features were simulated and compared to the compound data after learning Tone and Flash separately (see below).

### Machine Learning Methodology

To determine weights that would make the model fit the data we used the distribution of lever press responses across 100 time steps (20 pre stimulus, 80 during stimulus) to train the network to generate a 100 time step output activation vector that approximated the training vector. The weights that minimized the mean square error between the behavioral data and network output vectors were learned by utilizing the Levenberg–Marquardt algorithm from the Matlab Neural Net Toolbox to train the network. The training basically implements a back-propagation algorithm through time (BPTT) (Hagan et al., 1996; Haykin, 2004). This algorithm unfolds the RNN into a multilayered feed-forward network where each time epoch constitutes another layer (see Appendix A for a simplified description of the unfolding mechanism). Once the RNN is unfolded, multilayered back propagation with the Levenberg–Marquardt refinement can be used to identify the weights (see Table 2). As described above, saliency functions were also implemented between the Flash and Tone summation units, to better approximate the network’s performance to the Tone + Flash compound empirical data (see Table 2 for saliency constants).

**TABLE 2:** The model weights W_1_–W_6_ were learned from the final average Tone Response data.

**Model weights**		**Descriptor**	**Saliency weights**	
W_1_	0.2342	Tone Go Input weight	cutoff_a	−0.8632
W_2_	−0.1774	Tone No-Go Input weight		
W_3_	57.3471	Tone Go recurrent weight	cutoff_v	−0.2
W_4_	1.3228	Tone No-Go recurrent weight		
W_5_	9.7741	Tone Go Sum weight	Auditory Sum Operator Threshold	1.6
W_6_	11.3814	Tone No-Go Sum weight		
W_7_	0.00081236	Flash Go Input weight	Visual Sum Operator Threshold	0.85
W_8_	−0.000521	Flash No-Go Input weight		
W_9_	865.0088	Flash Go recurrent weight	Time to reach cutoff_a	27 time steps
W_10_	1.1229	Flash No-Go recurrent weight		
W_11_	0.9659	Flash Go Sum weight	Time to reach cutoff_ v	54 time steps
W_12_	1.6959	Flash No-Go Sum weight		
W_13_	0.1	Final Integrator Input Weight		
W_14_	0.9	Final Integrator recurrent weight		

*The model weights W_7_ – W_12_ were learned from the final average Flash Response data. The final integrator weights, W_13_ and W_14_ were chosen to have a reasonably long time constant that can be used to integrate the Tone and Flash Timing information from the previous layers. The Saliency weights were chosen to improve fits of the model to the data after the learning took place.*

### Machine Learning for Tone Trials

**FIGURE 6:**
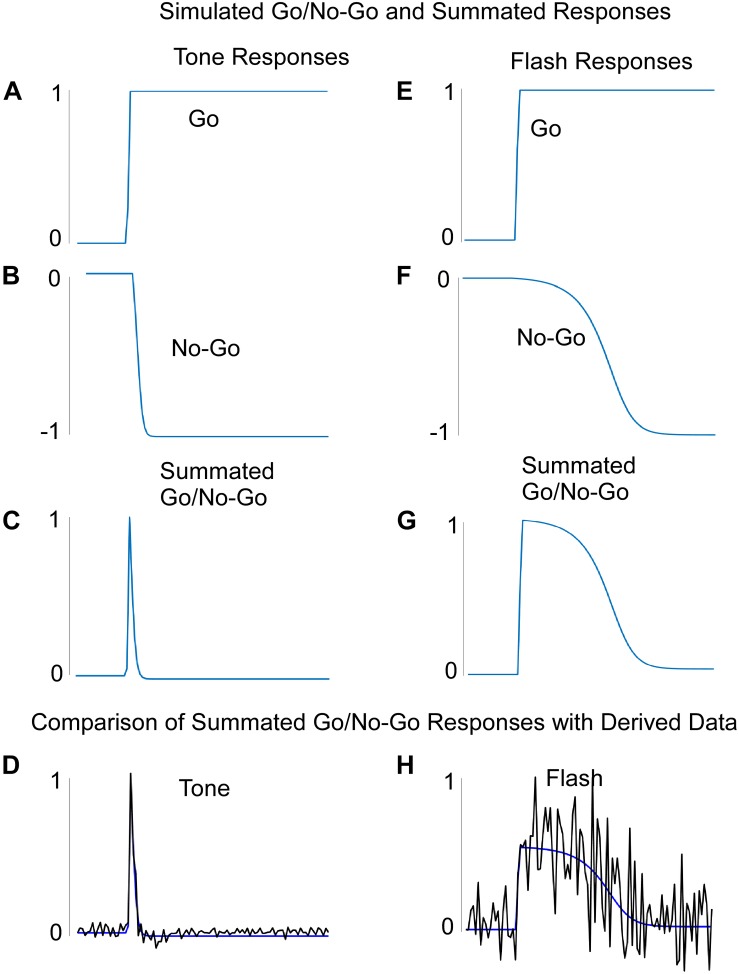
**(A–D)** The learned Go **(A)**, No-Go **(B)**, and summated Go/No-Go responses to a Tone input. The Summated Go/No-Go response compares favorably with the derived data for this response. **(E–H)** The learned Go **(E)**, No-Go **(F)**, and summated Go/No-Go **(G)** responses to a Flash input. The Summated Go/No-Go response compares favorably with the derived data for this response **(H)**. Note that the “derived” data was based on the values needed to produce the actual rodent empirical data when these were fed into the integrator unit.

**FIGURE 7:**
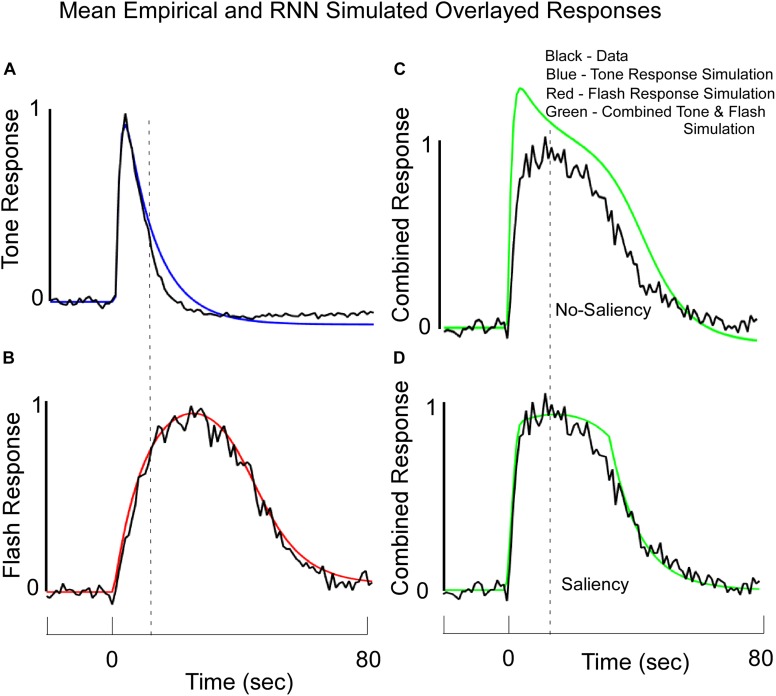
Average Normalized Response Functions observed on Tone **(A)**, Flash **(B)**, and Combined Tone + Flash probe trials in Block 11 **(C,D)** overlayed with the RNN model’s simulated data. Note, these simulated data reflect output values from the large fixed weight integrator. The model was trained to fit the empirical data for the Tone only and Flash only trials. **(C)** The fits to the data when there was no saliency and the Tone and Flash responses were linearly combined. **(D)** A stronger fit to the combined Tone + Flash data was obtained when we assumed that the simultaneous presentation of both stimuli diminished the “salience” of one stimulus on the other, but the learned weights in the network were not changed. These data support the idea that salience interactions among stimuli may be required to understand temporal averaging effects. See text for more details.

The system was first trained by keeping the final integrator and flash component weights fixed while also maintaining the flash input at zero. The data came from the non-reinforced probe trials with Tone in Block 11 of training (Figure 2A). Once these weights were learned, we inspected the output activation levels of the Go and No-Go recurrent units for each of the 100 steps on a simulated Tone trial. The Tone input generated a rapid rise in activation of the “Go” unit and a slower decrease in activation of the “No-Go” unit. Thus, the two counteracted one another over time (Figures 6A,B). The summated response (measured by the summation unit’s output activation level) was a pulse (Figure 6C), which was then used to drive the final integrator. When this summated function (Figure 6C) was applied to the integrator, the simulated tone response fit the data with great fidelity (Figures 6D, 7A). Because the flash input was zero, the flash component did not impact the response of the system to a pure tone.

### Machine Learning for Flash Trials

The network was similarly trained to respond to the Flash stimulus, but the Tone weights were kept constant as found during Tone learning. The final integrator weights were kept from learning as well and they were the same used for training Tone. The Go and No-Go units to a Flash input, respectively, also displayed a fast rise and slower fall in activation (Figures 6E,F). Notably, the No-Go unit decreased its output at a slower rate than on simulated Tone trials (Figures 6B,F). The Flash summation unit, which summed over its separate Go and No-Go units, showed a similar pulse to that of the Tone summation unit, but weights that were learned delayed the phase and increased the variance over time as compared to Tone (Figures 6C,G). When this widened pulse was applied to the final integrator whose weights were the same as for Tone input, the simulation fit the Flash data almost perfectly (Figures 6H, 7B). These simulations indicate that the Tone and Flash timing data could be generated separately by having different timing subsystems for the two stimuli that, nonetheless, combine at the final integration level.

### Machine Learning for Combined Tone + Flash Trials

After training the Tone and Flash separately (Figures 7A,B), the model was then tested to determine whether it would predict the combined response to Tone and Flash inputs. Without altering any of the weights and without incorporating any saliency functions, the model predicted a shift in phase when the inputs were combined (Figure 7C). It also predicted a rapid rise in responding to the compound stimulus, close to that seen to Tone (Figure 7A), as well as a slow decline in responding closely aligned to the Flash alone (Figure 7B). However, it did not accurately predict the shapes on these simulated compound trials. The peak was close to that of Tone (Compare Figure 7C with Figure 7A), and simulated responding to the compound stimulus overshot the empirical response function (Figure 7C). This discrepancy between model output and empirical data prompted us to consider a role that saliency might play in governing system performance. When we introduced the “saliency functions” for tone and flash components of the network described above, the empirical and simulated data fit with a much-reduced error (Figure 7D).

Because these parameters were non-linear functions, they could not be learned and the various saliency parameters of the effects of tone on flash and flash on tone were adjusted in a trial and error way. We did so in order to reduce the mean squared error between the empirical behavioral function on compound trials and the model’s response function on compound trials once parameters for Tone and Flash were learned.

Thus, the best predictor of the fit to the average compound data occurred when Tone was saturated by the interference of Flash, causing a rise in response close to that of tone and a saturation and cutoff of Flash and Tone to produce the decline in the compound response function. Tone interfered with the Flash internal signal at a time when the Flash No-Go signal was sufficiently negative (i.e., had surpassed a certain threshold). Conversely, the internal summed Go and No-Go Tone signal was cutoff when the Tone No-Go unit was sufficiently negative and surpassed a different threshold. Because the Flash signal rose to the threshold slower than the Tone, there was a non-symmetric non-linear interaction that governed the dynamic responses. The principle of Saliency that we have established is that while Tone and Flash can effectively interfere with the other, they are governed by different saturation and cutoff functions. The saturation level is determined by the presence of the other signal and the cutoff is determined by the No-Go component of the same signal. Both the saturation and cutoff are present only when both signals are processed simultaneously; otherwise, these units adopt a simple linear activation function (as was used on trials with one or the other stimulus present).

## Discussion

This study showed that a simple RNN, one that has local feedback, could simulate rodent data from a peak interval timing task. In particular, we demonstrated that the model could be trained to produce the different response functions we obtained with rats performing in a peak timing task when one stimulus (Tone) indicated reward availability after 5 s and a second stimulus (Flash) after 30 s. Further, we showed that the model approximated the behavior of the animals when probed with a compound stimulus (Tone + Flash). Specifically, both the rats and the model responded to the stimulus compound by “averaging” the two independent temporal estimates to reward such that maximal responding occurred at an interval intermediate between the two signaled by the individual stimuli (Swanton et al., 2009; Swanton and Matell, 2011; Matell and Henning, 2013; Matell and Kurti, 2014; Delamater and Nicolas, 2015; De Corte and Matell, 2016a, b).

The RNN neural network architecture presented in this study is unique in that it models interval timing using a small number of neurons to encapsulate the dynamic properties of interval timing. A feature of the model is that it converts a sensory step input into an appropriately timed pulse. This could be related to the observation that single cell neural firings in the dorsal striatum display temporal firing properties that match interval timing behavior (Matell et al., 2003). Furthermore, our model suggests that there should exist neurons that code the separate Go and No-Go components of the timing signal. This is in contrast to other RNN models that seek to derive properties of scalar timing directly from large populations of interconnected neurons (Simen et al., 2011; Luzardo et al., 2017; Hardy and Buonomano, 2018). Despite its simplicity, our model has some common conceptual features with other models interval timing. For example, SET (Church et al., 1994), the multiple-time-scale memory model of timing (Staddon and Higa, 1999; Staddon, 2005), spectral timing (Grossberg and Schmajuk, 1989; Buhusi and Oprisan, 2013), behavioral theories of timing (Killeen and Fetterman, 1988; Machado, 1997) and diffusion drift theories (Simen et al., 2011; Luzardo et al., 2017) all assume that integration or summation over time is an important component of temporal encoding. SET assumes that there is a pacemaker clock whose pulses are counted (i.e., integrated) over time, with response decisions obeying particular decision rules (Gibbon and Church, 1990). In this way, SET can be thought of as a dynamical system, perhaps operating at a more computational, rather than a mechanistic, level of analysis. Church and Broadbent (1990) suggested one way to implement SET’s key ideas within the framework of a feedforward connectionist network. Our approach differs from this and related models (Oprisan and Buhusi, 2011; Buhusi and Oprisan, 2013) by emphasizing the feedback components within a RNN. Multiple time scale, spectral timing, and behavioral theories of timing can be construed as assuming that the reinforced time is encoded as one component within a series of cascaded integrators, and that what is learned is the reinforced strengths, or values, of these different components. In particular, the component occurring maximally at the time of reward accrues maximal strength (also Pavlov, 1927). The RNN proposed in this study also utilizes the concept of an integrator, but we implement it as an individual node with a recurrent feedback loop whose weight determines its “time constant,” i.e., its rate of growth and decay. In this way, maximal responding arises when the summation unit reaches its peak, but, importantly, this is accomplished in a system that neither requires a pacemaker-clock system nor a cascade of separate temporally discriminable states. A main distinction between our RNN approach and those of many other approaches is that temporal memory is encoded in the connection weights of the RNN and the recurrent loop weights.

The apparent simplicity of the RNN proposed here is noteworthy. Each input stimulus converges to two separate recurrent units that, themselves, converge on a single summation unit that, ultimately, feeds into a single response integrator unit. Thus, each stimulus is assumed to be part of a 4-unit dynamical “timing” system that feeds into a response output integrator unit (see Figure 5). One important discovery with this network was the observation that when training it to learn a particular response function (e.g., to Tone, or to Flash), the first two recurrent units always adopted opposing behavioral functions. We label one of these the “Go” function and the other the “No-Go” function. In essence, the RNN learns by developing a tendency to respond (Go) and a separate tendency to turn off the response (No-Go).

Through the combination of these two behavioral tendencies, the network learns to appropriately time its output. In an additional analysis we used the data from each block of training (Figure 1) to train the network and we inspected the status of the Go and No-Go units. We observed that the RNN rapidly acquired the tendency to respond through a strong activation of the Go unit, but only more slowly developed the opposing tendency to turn off the response through increasing suppression of the No-Go unit. In other words, just like the animals, the RNN rapidly learned to respond, but only with additional training learned to withhold responding at inappropriate times. SET, and other approaches, interprets the loss of responding at extended times in terms of an increasing dissimilarity between a representation of elapsed time to the remembered reinforced time. Here, we assume that it reflects the combined influences of opposing learned response tendencies that appear to be acquired at different rates. It remains to be determined whether including more than two recurrent units at this stage of the RNN would impact any of the model’s predictions.

Another important issue regarding the RNN concerns how stochastic decision rules for responding can be incorporated. Our RNN model generates a deterministic output, which would have to be converted to a stochastic signal that could vary from trial to trial (or even within trial) (Church et al., 1994). This aspect of the model remains to be developed.

We earlier suggested that while many different types of models of interval timing can explain key aspects of interval timing data, all of those theoretical approaches generally have difficulty with the averaging phenomenon (Swanton et al., 2009; Swanton and Matell, 2011; Matell and Henning, 2013; Matell and Kurti, 2014; Delamater and Nicolas, 2015; De Corte and Matell, 2016a, b). We replicated that empirical effect here, but went on to show how it might arise from the RNN framework. In particular, we showed that the response functions to the Tone + Flash compound produce a response timing function whose peak and variance was between those seen to Tone and Flash when given separately. In order to better fit these data, we assumed that the activation functions at the stage of inputs to the final response integrator unit needed to be modified by what we refer to as non-linear “saliency functions.” In particular, we assumed that when the Tone and Flash stimuli were compounded, they mutually interfered with one another’s processing (though not necessarily to the same degree). In this model, saliency is defined as the conditions that must be met when two (or more) stimulus are present for one stimulus to interfere with or block the transmission of the other. There were two aspects of the saliency operation that we considered. A primary saliency operation in this model is the saturating of the summed Go/No-Go signal when another signal is present. Thus, one signal such as Flash transmission can limit the transmission of the Tone response. Another saliency operation is the blockage of signal transmission at a certain point, which is represented by the switch. For Tone transmission, for example, it is in the 0 position for NO blockage and in the 1 position when there IS blockage. In this model, the switch is in the 1 position (blockage) when there is another signal (Flash) present AND the No-Go of the Tone response is negatively greater than some cutoff. This is the purpose of the AND gate and switch. Together, this type of saliency is capable of fitting the nuances of the compound data, once the weights for Tone and Flash have been learned separately. While this may not be a unique methodology for implementing saliency, it does suggest that saliency may play an important role in refining the response to compound stimuli once weights have been learned for responding to stimuli separately. It opens the possibility of more in depth studies of saliency and its role in implementing event timing.

The specific rules for determining salience disruptions, more generally, have not been elucidated. Other research has shown that mutual interference can occur when two stimuli are equivalently salient, but that asymmetrical disruptions occur when one stimulus is stronger (e.g., Mackintosh, 1976). Relatedly, Matell and Kurti (2014) showed that temporal averaging varied as a function of the differential reward probabilities and stimulus modalities of the early and late time signals. Our suggestion is that these effects reflect differential salience disruptions on compound trials when stimuli are differentially salient to begin with or when different reinforcement probabilities are used. This mutual disruption of signal processing could be an important consideration when interpreting temporal averaging studies.

A key aspect of any theory of interval timing should address its scalar timing property (Gibbon, 1977; Gibbon et al., 1984; Wearden et al., 1997) as it has long been recognized as a fundamental issue in timing (Gibbon, 1977). We utilized standard methods (taken from Matell and Kurti, 2014) to compute the peak times and widths of our average Tone, Flash and Compound data to determine whether there was scaling across these trial types. We defined the CV as the width of the behavioral function/it peak time for Tone, Flash, and Compound trials and observed that they were not constant and, therefore, not in accordance with scalar invariance. It is not clear why our data did not obey the scalar timing principle, but it may be related to the specific short and long intervals used (5 s, 30 s).

The RNN model proposed in this study is based on the contribution of combinations of integrators to timing performance (Staddon and Higa, 1999; Staddon, 2002, 2005). Our starting point was to ask, first, if different behavioral timing functions could be modeled using the RNN approach. We show that the network weights are trainable so that trained weights can simulate data from tone trials whose peak occurs at 5 s as well as from flash trials whose peak occurs at 30 s and fits our data almost exactly. For example, a constant tone or constant flash input can be trained individually to respond to a certain rise and fall time. In the RNN model, the constant tone input generated a fast rising Go response and a slower rising No-Go response in the negative direction at the first processing layer. When these two responses were summated, a pulse was generated. Because the time constants of these components were trained using the empirical data, the pulse contains information about time of reward (the peak value). When this pulse is processed through an activation function, it scales and shapes the pulse that can be input to a final integrator, with a long rise time, whose output can be used by higher “cognitive” centers to decide whether or not to respond by bar pressing. We also showed that this final integrator can then be used to integrate compound stimuli whose timing is different from its individual components. What is perhaps intriguing about this model’s predictions is that we utilized the method of Matell and Kurti (2014) to compute the peak times, widths, and CVs for Tone, Flash and Compound data to determine whether there was scaling across these trial types. Although scalar invariance was not observed empirically, our model predicted these response functions with great fidelity. We suspect that different configurations of weights (within the networks “weight space”) may, indeed, produce response outputs that conform to scalar invariance. This was not our focus here, but the present framework does present the possibility in future developments that the model could differentiate when and under what conditions scalar invariance may or may not be present.

More generally, the kinds of dynamic properties of systems that combine integrators are ubiquitous across various sensorimotor systems. The vestibulo-ocular and optokinetic reflexes are governed by combinations of feedback control mechanisms that have equivalent dynamical responses as the RNN proposed in this study (Raphan et al., 1977, 1979; Fanelli et al., 1990). The concept of an integrator is at the core of the model of the vestibulo-ocular reflex (VOR) (Raphan et al., 1979; Raphan and Cohen, 1996, 2002). For example, when the head is rotated with a step in velocity, eighth nerve afferents respond in a pulsatile manner as a second order system with a rising time constants of 0.003 s and an opposing falling time constant of 4–5 s (Fernandez and Goldberg, 1971; Goldberg and Fernandes, 1975; Wilson and Melvill-Jones, 1979; Raphan et al., 1996; Dai et al., 1999). This has been modeled with control systems using integrators with feedback similar to the RNN models proposed in this study (Raphan et al., 1996; Dai et al., 1999). The feedback mechanisms at this level comes from the viscosity of the endolymph fluid in the canal and the elasticity of the cupula, in which is embedded the hair cells that drive the eighth nerve afferents that code the movement of the head (Wilson and Melvill-Jones, 1979). This model is similar to the summation of the Go and No-Go responses presented here.

An important contribution regarding VOR processing, is the presence of another integrator at the level of the vestibular nuclei in the brainstem that lengthens the time constant at the periphery to a longer time constant of about 12 s, seen in medial vestibular nuclei neurons (Waespe and Henn, 1977a, b, 1978) and in eye velocity responses (Raphan et al., 1979). This central integrator known as the velocity storage integrator is also accessed by the optokinetic system (Cohen et al., 1977), which then activates another integrator known as the velocity-position integrator (Skavenski and Robinson, 1973; Skavenski et al., 1981; Raphan and Cohen, 2002). The central feedback mechanisms are not local as they are in the mechanical feedback that occurs within semicircular canals. Rather, they appear to be more global and with projections across the commissure and back (Galiana et al., 1984; Wearne et al., 1997). These mechanisms were modeled using control theoretic concepts, which have the same architectural structure as the RNN proposed here, although there has been some work to model this integrator using neural nets (Anastasio, 1991). In addition, similar feedback control has been used to model locomotion reflexes (Raphan et al., 2001; Osaki et al., 2007, 2008) and an RNN with feedback has been used to model vestibulo-autonomic interactions (Raphan et al., 2016) to predict vaso-vagal responses as well as vaso-vagal syncope (Yakushin et al., 2016). This suggests that the central nervous system utilizes integrators to implement sensory motor transformations whose weights can be learned to adapt the behavior. It was of interest that the transformations that were utilized to model interval timing behavior were structured in the same manner as that for the VOR.

The RNN model proposed here has not addressed the important problem of identifying a trial-by-trial learning mechanism from the timing of the reward. Rather, we used the animals’ asymptotic empirical data to identify a set of weights that result in appropriate network output on simulated Tone and Flash trials, and then we used this to predict network performance on stimulus compound trials. The reward timing is therefore embedded in these empirical responses, which are mapped to the RNN weights. The RNN developed separate Go and No-Go functional units, and we adopted particular saliency functions (based on stimulus cross-coupling and No-Go threshold mechanisms) in order to account for the temporal averaging effect. Therefore, we have provided a ‘proof of concept’ that the RNN framework can be usefully applied to model interval timing data and showed that temporal averaging effects may arise from that network. More specifically, we have shown that weights of the RNN can be found so that the model is capable of faithfully reflecting the empirical data arising when one stimulus signals a 5 s and another a 30 s reward time. But in order to show this we used a somewhat arbitrary learning algorithm (found in MatLab’s toolbox) in conjunction with “teaching” signals provided by the animals’ actual response functions. This approach shows that, in principle, the RNN is capable of producing a set of weights between nodes that could give rise to scalar timing and temporal averaging effects. But to show that the RNN could learn this in a more realistic way requires specification of the learning mechanisms whereby separate timing functions can be learned in a food-reinforced learning situation. One approach is that provided by “reinforcement learning” (Sutton and Barto, 2018), which could be applied to dynamical systems learning. However, we are not aware of its application to RNNs, and this would need to be developed further. Regardless of the details of how this might be accomplished, however, we have identified a new model structure that could be extremely important for our understanding of how the central nervous system encodes interval timing.

## Data Availability

The datasets generated for this study are available on request to the corresponding author.

## Ethics Statement

All procedures used in the experiment with the rats were approved by the IACUC of Brooklyn College, and were in compliance with NIH guidelines as identified in the Guide for the Care and Use of Laboratory Animals, 8th Ed.

## Author Contributions

TR contributed to the overall conceptual framework for the study, and responsible for developing the model, and the organization and writing of the manuscript. ED contributed to the development of the implementation of the model in Matlab, writing the programs for comparison of model output with the data and identifying the weights to simulate the data, and writing of the manuscript. AD contributed to the experimental data, which was used as the database in the training of the model to determine the weights to simulate the data, writing of the manuscript, and conceptual development of the model.

## Conflict of Interest Statement

The authors declare that the research was conducted in the absence of any commercial or financial relationships that could be construed as a potential conflict of interest.

## Figures and Tables

**FIGURE A1:**
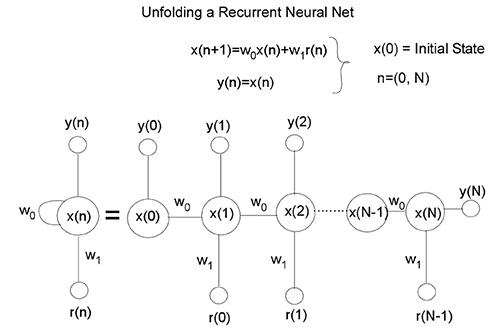
The unfolding of a one dimensional recurrent neural net over time. The Back propagation through time (BPTT) algorithm can be used to identify the weights.

## APPENDIX A

A recurrent neural net can be thought of as a feedforward network, in which the recurrent network is unfolded in time (Figure A1). If this network were to be trained over a 100 time steps, as is for the experiments modeled in this paper, we could unfold the network to create one 100 layers – one for each time step. The Back Propagation Through Time (BPTT) algorithm then represents a method for computing the gradients over these layers and this fixed network produces the same final result for the computed weights as the recurrent neural net with the same weights.

There are some problems with this type of learning, however. If a sigmoid transfer function is used, then if the output of the network is near the saturation point for any time point, the resulting gradient could be quite small and could impact the convergence. Another problem in training dynamic networks is the shape of the error surface. It has been shown that the error surfaces of recurrent networks can have spurious valleys that are not related to the dynamic system that is being approximated. The underlying cause of these valleys is the fact that recurrent networks have the potential for instabilities. However, it is possible, for a particular input sequence, that the network output can be small for a particular value greater than one in magnitude, or for certain combinations of values. Finally, it is sometimes difficult to get adequate training data for dynamic networks. This is because the inputs to some layers will come from tapped delay lines. This means that the elements of the input vector cannot be selected independently, since the time sequence from which they are sampled is generally correlated in time. Unlike static networks, in which the network response depends only on the input to the network at the current time, dynamic network responses depend on the history of the input sequence. The data used to train the network must be representative of all situations for which the network will be used, both in terms of the ranges for each input, but also in terms of the variation of the inputs over time.

Static multilayer networks can be used to approximate functions. Dynamic networks can be used to approximate dynamic systems. A function maps from one vector space (the domain) to another vector space (the range). A dynamic system maps from one set of time sequences (the input sequences) to another set of time sequences (the output sequences). For example, the network of Figure A1 is a dynamic system. It maps from input sequences to output sequences. The BPTT algorithm starts from the last time point and works backward in time. In addition to the gradient, versions of BPTT can be used to compute Jacobian matrices, as are needed in the Levenberg–Marquardt algorithm. Once the gradients or Jacobians are computed, many standard optimization algorithms can be used to train the networks. The BPTT algorithm, however, usually requires more memory storage and for large networks, the memory requirements would be unmanageable. Despite the shortcomings of the BPTT algorithm, the recurrent neural network that we used has a small number of interconnected units and therefore, the BPTT algorithm using the Levenberg–Marquardt algorithm in the Matlab neural Net Package worked quite well in converging to weights that fit the data and gave insight into the internal workings of the timing generator.
